# Dementia and COVID-19: The Ones Not to Be Forgotten

**DOI:** 10.1177/1533317520947505

**Published:** 2020-08-19

**Authors:** Duarte Barros, Flávia Borges-Machado, Óscar Ribeiro, Joana Carvalho

**Affiliations:** 1 CIAFEL, Research Center in Physical Activity, Health and Leisure, Faculty of Sport, 26706 University of Porto, Portugal; 2 CINTESIS, Center for Health Technology and Services Research, 451616 Department of Education and Psychology, University of Aveiro, Portugal

**Keywords:** SARS-COV-2, neurocognitive disorder, pandemic, caregiving

Humankind has been dealing with a devastating colossal pandemic, described as the greatest challenge of the 21st century in health and social care – dementia. Worldwide it is estimated that every 3 seconds someone develops dementia. This syndrome prevalence is not slowing down and already more than 50 million people live with it, a number that is expected to increase to 152 million in 2050.^1^ Supporting this data, Alzheimer Europe estimated that almost 10 million people live with dementia in Europe. Portugal is alarmingly one of the most affected countries and will exceed the European trend by doubling its current prevalence to 3.82% of the total population until 2050.^2^

Presently, we are facing another pandemic threat due to COVID-19, a highly infectious disease that rapidly spread around the globe. The first 2 cases of COVID-19 in Portugal were confirmed on 2 March 2020. On 12 March, the Portuguese Council of Ministers approved a set of extraordinary measures like closing schools, nurseries, kindergarten, and daycare centers to restrict social contact and group activities. The state of alert was declared on 13 March 2020. Following the exponential increase of cases, the Portuguese government declared the state of emergency and mandatory lockdown from 18 March till 2 May. Therefore, very strict measures of physical-social distancing were adopted to control the outbreak, which in itself represents a physiological and cognitive challenge with significant repercussions on health and wellbeing.^3^

People with dementia (PwD) and their relatives’ lives were highly impacted by these measures because many group activities and care-related services were canceled and visitors were prohibited in nursing homes for 3 months. Most day-care centers were closed on March 16th for an undetermined period without specific guidelines or recommendations, imposing this population to stay at home without premeditated support care.^4^ Despite no official data is available on the prevalence of dementia across the various settings in Portugal, it is estimated that this population was highly impacted since nearly 31% of participants in the daycare center and almost 50% of residents in the nursing homes might have dementia.^5^

Such a drastic shift in their lives and enforced physical inactivity are expected to lead to a significant worsening of their cognitive and functional status,^6^ exacerbation of preexisting neuropsychiatric symptoms, and onset of new disruptive behaviors. As a consequence, this snowball effect adds more strain to already-highly levels of burden and exhaustion of their familiar relatives, especially those who have become full-time carers and/or are in remote working.^7^

Relentlessly, prominent organizations have raised awareness, highlighting the need to support PwD and their carers during this health crisis. Paradoxically, these populations hit by 2 pandemics at the same time have received little interest from the scientific community. By 02 June 2020, 17181 articles related to COVID-19 were found on Pubmed, and from those only 33 (0.19%) concerned dementia.

COVID-19 emergency should not overshadow dementia care. Neglecting it may result in the rapid rise of severe cases, disability numbers, and institutionalizations. Moreover, not ensuring the required support to carers will have a detrimental effect on the dyads’ mental health. Particularly for Portugal, this health crisis uncovered how fragile the dementia paradigm is: in 27 June 2020, nearly 86% of the total deaths (n = 1346) from COVID-19 were in people aged 70 or more, and 40% of the total deaths were in nursing homes settings.^8^ Yet, it is unknown how many of these fatalities were PwD.

We expect that COVID-19 impact will be deep and long-lasting. As we are entering the post-emergency phase, it is urgent to determine the impact of COVID-19 on parameters that influence the disease’s trajectory and quality of life of both PwD and their carers.^7^ Additionally, it is an opportunity to improve the dementia care paradigm in Portugal, make research investments in home-based caregiving approaches and technology (e.g., telehealth, online training and support) to ensure that these vulnerable populations are not left behind in what regards their needs during upcoming crises.
